# The indole compound MA-35 attenuates tumorigenesis in an inflammation-induced colon cancer model

**DOI:** 10.1038/s41598-019-48974-9

**Published:** 2019-09-04

**Authors:** Keigo Kanehara, Shinobu Ohnuma, Yoshitake Kanazawa, Keisuke Sato, Shoji Kokubo, Hideyuki Suzuki, Hideaki Karasawa, Takehiro Suzuki, Chitose Suzuki, Takeshi Naitoh, Michiaki Unno, Takaaki Abe

**Affiliations:** 1 Department of Surgery, Tohoku University Graduate School of Medicine, Sendai, 980-8574 Japan; 2 Department of Gastroenterology, Tohoku University Graduate School of Medicine, Sendai, 980-8574 Japan; 3 Division of Nephrology, Endocrinology, and Vascular Medicine, Tohoku University Graduate School of Medicine, Sendai, 980-8574 Japan; 4 Department of Medical Science, Tohoku University Graduate School of Biomedical Engineering, Sendai, 980-8574 Japan; 5 Department of Clinical Biology and Hormonal Regulation, Tohoku University Graduate School of Medicine, Sendai, 980-8574 Japan

**Keywords:** Colon cancer, Gastrointestinal models

## Abstract

In inflammatory bowel disease, chronic inflammation results in the development of colon cancer known as colitis-associated cancer. This disease is associated with tumor necrosis factor-α (TNF-α) signaling. In addition, intestinal fibrosis is a common clinical complication that is promoted by transforming growth factor β1 (TGF-β_1_). In our previous study, MA-35 attenuated renal fibrosis by inhibiting both TNF-α and TGF-β_1_ signaling. This study aimed to identify the possible antitumor effects and antifibrotic effects of MA-35 using an AOM/DSS mouse model. MA-35 was orally administered every day for 70 days in the AOM/DSS mouse model. There was no difference in weight loss between the AOM/DSS group and the AOMDSS + MA-35 group, but the disease activity index score and the survival rate were improved by MA-35. MA-35 blocked the anemia and shortening of the colon induced by AOM/DSS. MA-35 reduced the macroscopic formation of tumors in the colon. In the microscopic evaluation, MA-35 reduced inflammation and fibrosis in areas with dysplasia. Furthermore, the TNF-α mRNA level in the colon tended to be reduced, and the interleukin 6, TGF-β_1_ and fibronectin 1 mRNA levels in the colon were significantly reduced by MA-35. These results suggested that MA-35 inhibited AOM/DSS-induced carcinogenesis by reducing inflammation and fibrosis.

## Introduction

Inflammatory bowel disease (IBD), such as ulcerative colitis (UC) and Crohn’s disease (CD), is clinically characterized by dysregulated intestinal inflammation. A serious long-term complication of chronic inflammation is the development of colon cancer, known as colitis-associated colorectal cancer (CAC), which is the major cause of death^1^. UC increases the cumulative risk of CAC by up to 18%, while that of CD reaches 8.3% after 30 years of disease^1–3^. Although the precise pathological mechanism of IBD is still controversial, inflammation is one of the most common potential pathogens of carcinogenesis in the colon. The underlying inflammation generates tumorigenesis and cancer development^4^. Recently, findings have demonstrated that the damaged epithelia and activated immune cells in the inflamed mucosa have an important role in the pathogenesis^5–7^. In addition, recent works suggest that a major factor in the inflammatory processes involves activated nuclear factor-kappa B (NF-κB) pathway via pro-inflammatory cytokines, tumor necrosis factor (TNF-α), interleukin (IL)-1, IL-6^8–10^. Persistent NF-κB activation in the epithelial cells has been suggested to contribute to the development of CAC^11^. Thus, earlier intervention of potent anti-inflammatory agents could be an effective way to prevent CAC.

An animal model of CAC with the combination of azoxymethane (AOM), a colonic genotoxic carcinogen, and dextran sulfate sodium (DSS), an inducer of colitis, has been widely used for analyzing the mechanism of the development and prevention of CAC^12,13^. All mice treated with this protocol developed tumors in the distal to middle colon. Several investigators demonstrated TNF-α inhibition in the same animal model prevents the development of CAC^14,15^. Moreover, TNF-α-deficient mice developed fewer tumors than WT mice^16,17^. Thus, it is considered that TNF-α plays a key role in the development of CAC. In addition, intestinal fibrosis is a common and devastating outcome in patients with IBD, and is associated with significant morbidity and mortality caused by bowel strictures and stenosis^18–20^.

Mitochonic acid 35 (MA-35), 5-(3,5-dimethoxybenzyloxy)-3-indoleacetic acid is a derivative of indole-3-acetic acid (IAA), a plant hormone auxin^21^. IAA is synthesized in the mouse liver and kidney^22^ and intestinal anaerobes^23^. IAA regulates growth and essential for plant’s life cycle and body development. In addition, IAA increased the growth of mouse and human fibroblasts^24^. We recently found that MA-35 showed anti-TNF-α activity mediated by inhibiting IκB kinase (IKK) phosphorylation, which attenuated the hepatic and renal inflammation in the mice^25^.

In addition, MA-35 concurrently showed an anti-TGF-β_1_ effect by inhibiting Smad3 phosphorylation and reducing the Smad3-driven expression of fibrotic genes. These dual blockades of TNF-α and TGF-β_1_ attenuated inflammation and renal fibrosis^25^. Because TNF-α plays a key role in the development of CAC, we examined the anti-tumor effect of MA-35 by inhibiting TNF-α signaling in AOM/DSS mouse model. The anti-fibrotic effect of MA-35 by inhibiting TGF-β_1_ signaling was also examined.

## Results

### MA-35 inhibited drug-induced colitis and the progression of colitis-associated colorectal cancer (CAC) in an AOM/DSS mouse model

**Figure 1:**
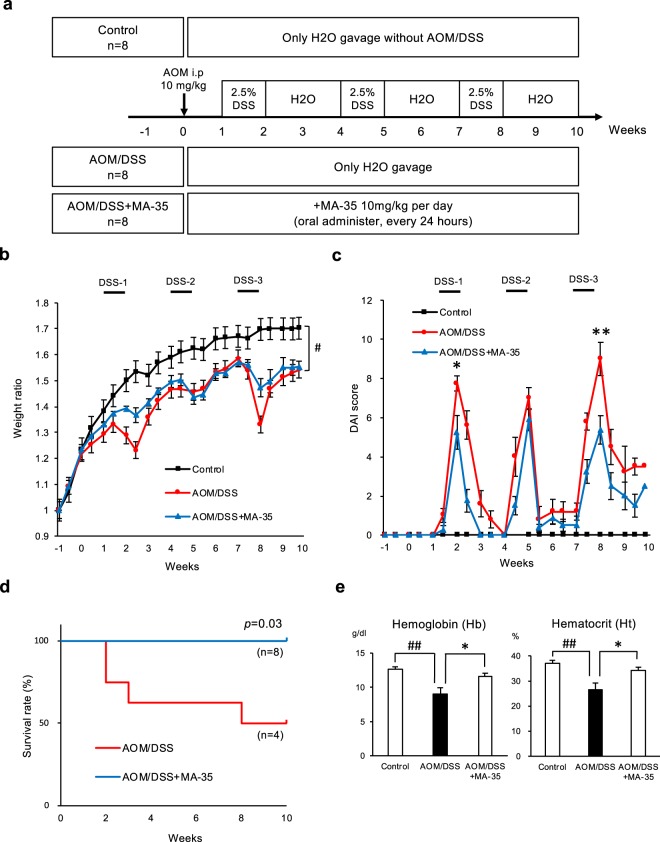
The effects of MA-35 on the AOM/DSS mouse model. (**a**) Experimental design. (**b**) Body weight loss observed in AOM/DSS-treated mice compared to the control group was significant. Administration of MA-35 (AOM/DSS + MA-35 group) did not alleviate body weight loss in the AOM/DSS group. (**c**) The DAI score of the AOM/DSS + MA-35 group was improved compared to the AOM/DSS group at weeks 2 and 8. (**d**) The survival rate of the AOM/DSS + MA-35 group was higher than that of the AOM/DSS group (*p* = 0.03). (**e**) In the AOM/DSS + MA-35 group, Hb and Ht were recovered compared to the AOM/DSS group. Data are presented as the mean ± SEM. Statistical analysis was performed by Tukey’s test and the log-rank test using the Kaplan-Meier method. ^#^*p* < 0.05, ^##^*p* < 0.01 versus the control group, *p < 0.05, **p < 0.01 versus the AOM/DSS group.

The CAC mouse model was established with procarcinogen AOM injection, followed by three cycles of the proinflammatory agent 2.5% DSS by oral administration. The study protocol is summarized in Fig. 1a. Because MA-35 had no influence on body weight, liver weight, liver histology, serum ALT, and serum TNF-α in our previous study^25^, we hypothesized that the influence of MA-35 treatment alone may be negligible in mice. We did not evaluate the MA-35 treatment alone group. As shown in Fig. 1b, the body weight was significantly reduced in AOM/DSS-treated mice compared to the control group. Administration of MA-35 to the AOM/DSS mice (AOM/DSS + MA-35 group) had no effect on the final body weight. However, the AOM/DSS group had a trend of decreased body weight compared with the AOM/DSS + MA-35 group (Fig. 1b). During the experiment, 4 mice in the AOM/DSS group died, probably due to the toxic effect of DSS (Fig. 1d). Therefore, we were unable to demonstrate a statistically significant difference in body weight between the groups due to the limited number of surviving mice in the AOM/DSS group.

Next, we measured the Disease Activity Index (DAI) score by sequential monitoring, as shown in Fig. 1c. After drinking DSS, AOM-treated mice exhibited signs of weight loss, diarrhea and bloody stool, resulting in an increase in the DAI score compared with that of the control group. Under this condition, the DAI score of the AOM/DSS + MA-35 group was significantly decreased compared with that of the AOM/DSS group at weeks 2 and 8 (*p* < 0.05, Fig. 1c).

We also examined the survival rate of these models. As shown in Fig. 1d, the survival rate of the AOM/DSS group was decreased, but that in the AOM/DSS + MA-35 group was significantly increased compared to the AOM/DSS group (*p* = 0.03). These data suggest that MA-35 had beneficial effects in ameliorating symptoms and increased longevity in the AOM/DSS colitis model.

We next performed biochemical and histological analyses in this model. Table 1 shows the biochemical parameters (Na, K, Cl, ionized calcium (iCa), total carbon dioxide (tCO2), blood urea nitrogen (BUN), hematocrit (Ht), hemoglobin (Hb), aspartic aminotransferase (AST), alanine transaminase (ALT)) at sacrifice. Among the data, Hb and Ht in the AOM/DSS group were significantly decreased compared with those in the control group (*p* < 0.01, Table 1 and Fig. 1e). Under this condition, the drug-induced reduction of Hb and Ht was significantly recovered in the AOM/DSS + MA-35 group (*p* < 0.05). Because gastrointestinal bleeding due to DSS administration^26^ was reported, these results suggest that MA-35 ameliorated anemia in experimental colitis.

**Table 1:** Biochemical parameters at the time of sacrifice. Data are presented as the mean ± SEM.

		Control	AOM/DSS	AOM/DSS + MA-35
Na	mmol/L	139.8 ± 0.8	140.0 ± 1.5	141.9 ± 1.3
K	mmol/L	7.8 ± 0.3	8.1 ± 0.2	7.8 ± 0.2
Cl	mmol/L	114.3 ± 0.8	114.0 ± 0.9	113.8 ± 1.2
iCa	mmol/L	1.1 ± 0.02	1.1 ± 0.02	1.1 ± 0.03
Total CO2	mmol/L	20.5 ± 0.9	21.8 ± 1.1	23.4 ± 0.6
BUN	mg/dL	23.8 ± 1.1	23.3 ± 2.9	18.4 ± 1.1
Ht	%	36.5 ± 1.0	26.5 ± 2.7^##^	34.1 ± 1.3^*^
Hb	g/dL	12.4 ± 0.3	9.0 ± 0.9^##^	11.6 ± 0.5^*^
AST	U/I	110.4 ± 12.7	139.3 ± 29.5	142.8 ± 14.7
ALT	U/I	51.0 ± 4.3	55.3 ± 9.0	51.6 ± 3.8

Statistical analysis was performed by Tukey’s test. ^#^*p* < 0.01 versus the control group, ^*^p < 0.05 versus the AOM/DSS group.

^##^*p* < 0.01 versus control group. ^*^p < 0.05 versus AOM/DSS group.

**Figure 2:**
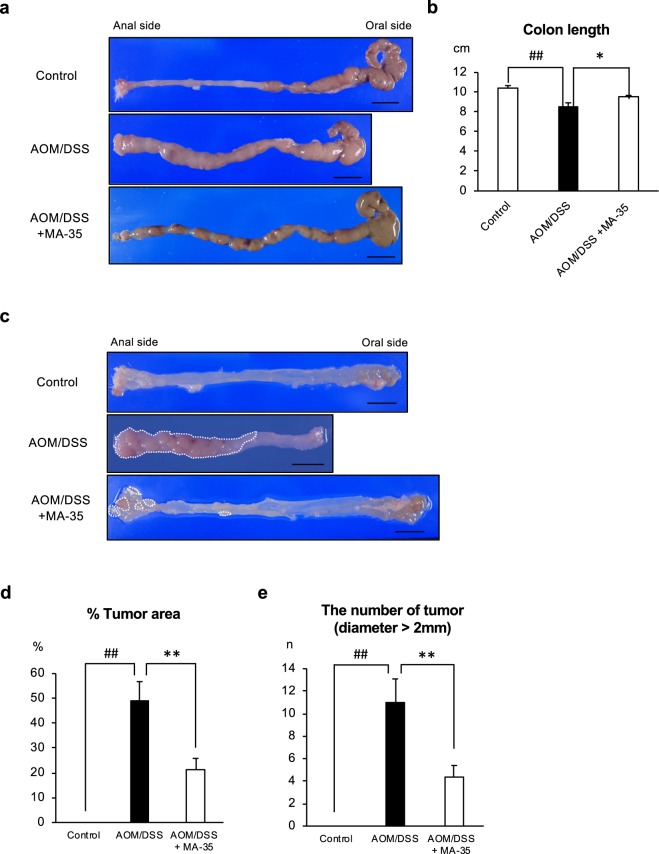
(**a**) Macroscopic view of the whole colon. The scale bar indicates 1 cm. (**b**) The colon length in the AOM/DSS + MA-35 group was significantly longer than that in the AOM/DSS group. (**c**) The macroscopic view of the colon lumen. Tumors developed in the distal colon of the AOM/DSS-treated mice (**surrounded by white line**). (**d**) The percentage of the tumor area was significantly decreased in the AOM/DSS + MA-35 group compared with the AOM/DSS group. (**e**) The number of tumors with diameters greater than 2 mm was significantly decreased in the AOM/DSS + MA-35 group compared to the AOM/DSS group. Data are presented as the mean ± SEM. Statistical analysis was performed by Tukey’s test. ^##^*p* < 0.01 versus the control group, *p < 0.05, **p < 0.01 versus the AOM/DSS group.

To confirm this, we next performed macroscopic examination in the colon. In mice that received DSS, shortening of the colon length is one marker for evaluating the severity of colonic inflammation^12,27–29^. Figure 2a shows representative images of the colon. The colon length of the AOM/DSS group was significantly decreased compared to that of the control group. However, administration of MA-35 abolished the DSS-induced shortening of the colon (*p* < 0.05) (Fig. 2b).

In this model, tumor development in the distal colon was reported previously^12,13^. Figure 2c shows a representative image. In the control group, there was no tumor area. In the AOM/DSS group, 49 ± 7.7% of the area was replaced by the tumor area. Under these conditions, the percentage (%) of the tumor area in the AOM/DSS + MA-35 group was significantly decreased compared with that of the AOM/DSS group (Fig. 2d). Furthermore, the number of tumors that were over 2 mm in diameter in the AOM/DSS + MA-35 group was also decreased compared to that of the AOM/DSS group (Fig. 2e). These data suggested that the tumor development induced by AOM/DSS treatment was reduced by MA-35.

**Figure 3:**
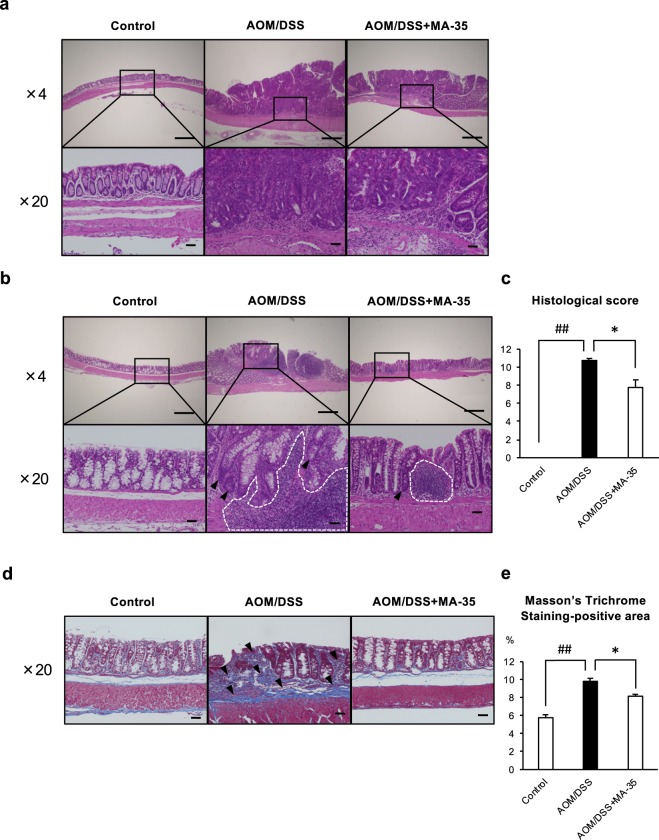
Histopathological analysis in the distal colon with H-E staining, middle colon to proximal colon with H-E staining and middle colon to proximal colon with Masson’s trichrome staining. (**a**) In the distal area, the infiltration of inflammatory cells and epithelial atypia were not different between the AOM/DSS and AOM/DSS + MA-35 groups. (**b**) In the middle colon to proximal colon, crypt distortion (**arrowhead**) and inflammatory cell infiltration (**surrounded by white line**) were significant in the AOM/DSS group compared with the control group. (**c**) The histological scores of the AOM/DSS + MA-35 group were significantly reduced compared to the AOM/DSS group. (**d**) In middle colon to proximal colon with Masson’s trichrome staining. In the AOM/DSS group, AOM/DSS induced severe fibrosis in the stroma of the colon (**arrowhead**). (**e**) The AOM/DSS + MA-35 group showed a significant reduction in the positive area compared to the AOM/DSS group. Scale bars of ×4 objective and ×20 objective were 500 μm and 50 μm, respectively. Data are presented as the mean ± SEM. Statistical analysis was performed by Tukey’s test. ^##^*p* < 0.01 versus the control group. *p < 0.05 versus the AOM/DSS group.

We next performed histological analysis in the distal colon (Fig. 3a) and middle colon to proximal colon (Fig. 3b) with H-E staining. In the AOM/DSS mouse model, inflammation and adenocarcinoma were observed mainly in the distal colon^12,13,29,30^. We then evaluated inflammation and atypia in the distal colon (Fig. 3a). In both the AOM/DSS-treated group and AOM/DSS + MA-35 group, adenocarcinoma was mainly detected in the distal colon (Fig. 3a). In the cancer area of the distal colon, infiltration of inflammatory cells and epithelial atypia were not different between the AOM/DSS group and the AOM/DSS + MA-35 group (Fig. 3a).

We next examined the section from the middle colon to the proximal colon. **Fi**. **3b** shows a representative image of the middle to proximal colon with H-E staining. In these sections, crypt distortion (Fig. 3b, **arrowhead**) and inflammatory cell infiltration (Fig. 3b, **surrounded by white line**) were significant in the AOM/DSS group compared with the control group. The inflammation score (histological score)^31,32^ was also increased (Fig. 3c**)**. In contrast, the histological score was significantly decreased in the AOM/DSS + MA-35 group (Fig. 3c**)**. Furthermore, to evaluate fibrosis in the middle to proximal colon, Masson’s trichrome staining was also performed (Fig. 3d,e**)**. AOM/DSS induced severe fibrosis in the stroma of the colon (Fig. 3d, **arrowhead**). Under this condition, MA-35 significantly reduced the Masson’s trichrome-positive area in the AOM/DSS + MA-35 group (Fig. 3d,e**)**, suggesting that MA-35 not only inhibited inflammation but also fibrosis induced by AOM/DSS.

**Figure 4:**
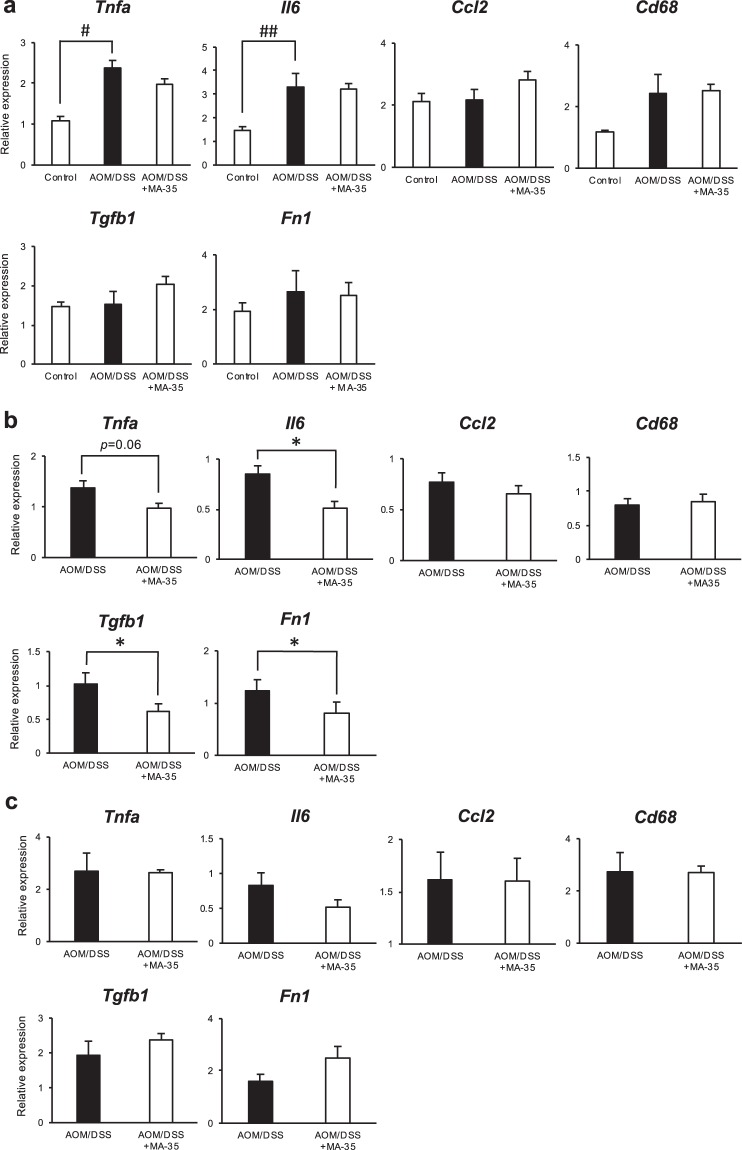
The mRNA levels of *Tnfa*, *Il6*, *Ccl2*, *Cd68*, *Tgfb1*, and *Fn1* were measured by real-time RT-PCR. (**a**) In the normal tissue of the proximal colon, the expression of *Tnfa* and *Il6* mRNA levels in the AOM/DSS group were significantly higher than those in the control group. (**b**) In the dysplasia tissue of the middle colon, MA-35 caused a significant reduction of the *Il6*, *Tbfb1*, and *Fn1* mRNA levels and tended to reduce the *Tnfa* mRNA levels. (**c**) In the cancer tissue of the distal colon, there were no significant differences between the two groups. Data are presented as the mean ± SEM. Statistical analysis was performed by Tukey’s test or Student’s *t*-test. ^#^*p* < 0.05, ^##^*p* < 0.01 versus the control group. *p < 0.05 versus the AOM/DSS group.

To clarify the anti-inflammatory and antifibrotic effects of MA-35, the mRNA expression levels of inflammatory genes (tumor necrosis factor-α (*Tnfa)*, interleukin-6 (*Il6)*, monocyte chemotactic protein-1 (MCP-1/*Ccl2*), the cluster of differentiation 68 (*Cd68*)) and fibrotic genes (transforming growth factor beta 1 (*Tgfb1*) and fibronectin 1 (*Fn1*)) in the proximal colon (Fig. 4a), middle colon (Fig. 4b) and distal colon (Fig. 4c) were examined by real-time RT-PCR.

In the proximal area (Fig. 4a), the expression levels of *Tnfa* and *Il6* in the AOM/DSS group were significantly higher than those in the control group, suggesting drug-induced inflammation, although there was no macroscopic dysplasia or cancer in the area. Other cytokines (*Ccl2* and *Cd68*) or fibrotic genes (*Tgfb1 and Fn1*) were not changed in the AOM/DSS group compared with the control.

We next examined gene expression in the middle colon, which showed dysplasia with different pit patterns and thicknesses^33^. The expression level of *Tnfa* in the AOM/DSS + MA-35 group showed a stronger tendency to decrease, and the expression of *Il6* was significantly decreased in the AOM/DSS + MA-35 group compared with that of the AOM/DSS group (Fig. 4b). In addition, the expression levels of *Tgfb1* and *Fn1* were significantly decreased compared with those of the AOM/DSS group. In the distal colon (Fig. 4c), we examined the cancer tissue induced by AOM/DSS. In this area, there was no significant difference in the inflammatory and fibrotic genes (Fig. 4c). These data suggest that MA-35 may inhibit AOM/DSS-induced carcinogenesis by reducing inflammation following fibrosis in the colon with dysplasia.

### MA-35 suppresses the TNF-α pathway by inhibiting IKK phosphorylation and the TGF-β_1_ pathway by inhibiting Smad2/3 phosphorylation

**Figure 5:**
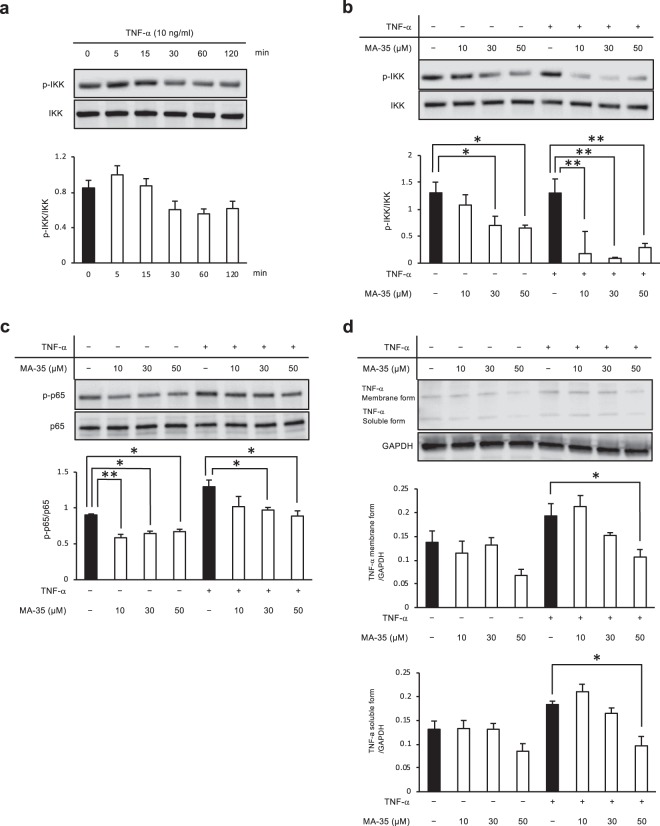
Effect of MA-35 on TNF-α-induced IKK phosphorylation in HT-29 cells. (**a**) Serum-starved HT-29 cells were stimulated with TNF-α (10 ng/ml). After 5 min, p-IKK was maximized. (**b**) Serum-starved HT-29 cells were preincubated for 60 min with or without MA-35 (10 μM, 30 μM, 50 μM) and then stimulated with TNF-α (10 ng/ml) for 5 min. MA-35 reduced the phosphorylation of IKK with or without TNF-α stimulation. (**c**) MA-35 reduced the phosphorylation of NF-κB p65 with or without TNF-α stimulation. (**d**) MA-35 reduced TNF-α with TNF-α stimulation. The grouping of blots cropped from different gels. Intact western blot results can be seen in Supplementary Information. Data are presented as the mean ± SEM. Statistical analysis was performed by Dunnett’s test. *p < 0.05, **p < 0.01.

**Figure 6:**
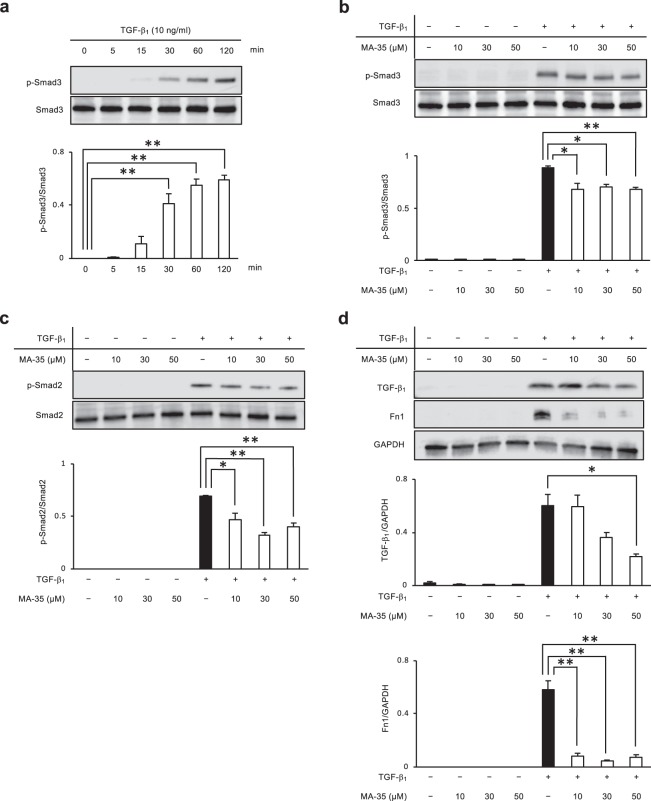
Effect of MA-35 on TGF-β-induced Smad3 phosphorylation in HT-29 cells. (**a**) Serum-starved HT-29 cells were stimulated with TGF-β_1_ (10 ng/ml). After 120 min, p-Smad3 was maximized. (**b**) Serum-starved HT-29 cells were preincubated for 60 min with or without MA-35 (10 μM, 30 μM, 50 μM) and then stimulated with TGF-β_1_ (10 ng/ml) for 120 min. MA-35 at 10 μM, 30 μM and 50 μM reduced the phosphorylation of Smad3, which induced TGF-β_1_. (**c**) MA-35 at 10 μM, 30 μM and 50 μM reduced the phosphorylation of Smad2, which induced TGF-β_1_. (**d**) MA-35 at 50 μM reduced TGF-β_1_ and MA-35 at 10 μM, 30 μM and 50 μM reduced Fn1, which induced TGF-β_1_. The grouping of blots cropped from different gels. Intact western blot results can be seen in Supplementary Information. Data are presented as the mean ± SEM. Statistical analysis was performed by Dunnett’s test. *p < 0.05, **p < 0.01.

During inflammation, the TNF-α released from macrophages binds to its membrane TNF receptor and then phosphorylates IκB kinase (IKK). This phosphorylated IKK induces degradation of the inhibitory binding protein of nuclear factor-kappa B (NF-κB) p65/p50, termed “IκB”, which is followed by translocation of the released NF-κB p65/p50 heterodimer into the nucleus. This nuclear NF-κB p65 translocation regulates the expression of various proinflammatory genes, such as TNF-α, MCP-1 and IL-6^34^. TNF-α production further induces the activation of the IKK/NF-κB pathway and forms a positive feedback loop^35^. In our previous study, we reported that MA-35 inhibited TNF-α/IKK signaling and TGF-β_1_/Smad3 signaling in the human hepatic stellate cell line LX-2 cells and rat kidney NRF-49F fibroblasts^25^. Therefore, we examined the effect of MA-35 on the TNF-α signal transduction pathway and TGF-β_1_ pathway using the human colon cancer cell line HT-29. As shown in Fig. 5a, the phosphorylation of IKK was maximally increased 5 min after TNF-α stimulation in the colon cancer cell line HT-29. Under these conditions, IKK phosphorylation was significantly reduced by MA-35 compared with the control (Fig. 5b). Next, we analyzed the expression of both NF-κB p65 and TNF-α as the downstream signals for p-IKK. MA-35 significantly inhibited the phosphorylation of NF-κB p65 (Fig. 5c) and TNF-α (Fig. 5d). We also examined the effect of MA-35 on the TGF-β_1_ signaling pathway. TGF-β_1_-activated Smad3 phosphorylation (Ser423/425) accelerates the translocation of the Smad2/3-Smad4 heterotrimer complex to the nucleus and facilitates the transcription of TGF-β_1_ target genes^36^. We examined the human colon cancer cell line HT-29, which showed the phosphorylation of Smad3 after TGF-β_1_ stimulation. The phosphorylation of Smad3 was maximally increased 120 min after TGF-β_1_ stimulation in the colon cancer cell line HT-29 (Fig. 6a). Under these conditions, TGF-β_1_ markedly induced Smad3 phosphorylation, and this upregulated Smad3 phosphorylation was significantly inhibited by MA-35 (Fig. 6b). In addition, TGF-β_1_-induced Smad2 phosphorylation was significantly inhibited by MA-35, suggesting that MA-35 inhibits not only Smad3 but also Smad2 (Fig. 6c). We further analyzed the expression levels of Fn1 and TGF-β_1_ as the downstream signals for p-Smad2/3. As a result, MA-35 significantly inhibited the expression of Fn1 and TGF-β_1_ (Fig. 6d). These data further suggest that MA-35 inhibited both inflammation and fibrosis through the IKK and Smad2/3 pathways, respectively.

## Discussion

In the present study, we showed that the indole derivative compound MA-35 inhibited AOM/DSS-induced carcinogenesis by reducing inflammation and fibrosis in the colon through inhibitory effects on both the TNF-α pathway by NF-κB and the TGF-β_1_ pathway by Smad2/3. NF-κB is activated via the phosphorylation of IKK^34^, and a previous study reported that metformin, a biguanide derivative, attenuated IκBα phosphorylation and NF-κB DNA-binding activity, resulting in the attenuation of acute murine colitis, chronic colitis and colitis-associated tumorigenesis in mice^37^. Therefore, the direct inhibition of IKK phosphorylation has a key role in the anti-inflammatory effect and antitumorigenic effect of MA-35. MA-35 also exerts anti-TGF-β/Smad signaling by inhibiting Smad3 phosphorylation (Fig. 6b). TGF-β_1_ plays a unique and pivotal role in homeostasis, wound healing, fibrosis, angiogenesis, carcinogenesis and differentiation of the cell^38–40^, and alterations in the TGF-β_1_ signaling pathway promote cancer cell growth and influence the cancer biological behavior^40,41^. Recently, Smad2/3 phosphorylation was shown to be an important event in CAC and can serve as a biomarker for CAC^42^, and Smad phosphorylation levels were also higher in the colon of AOM/DSS-treated mice^43^. Thus, the inhibitory effect of MA-35 on Smad3 phosphorylation may be related to the antitumorigenic effect.

We also found that MA-35 reduced fibrosis in the colon (Figs 3e and 4b). Intestinal fibrosis is a common and devastating outcome in patients with IBD and is associated with significant morbidity and mortality^18–20^. TGF-β_1_ is known to promote the transcription of fibrosis-associated molecules, such as collagens^44^, and leads to the development of intestinal fibrosis. The serum levels of TGF-β_1_ and its mRNA expression levels are also known to increase in patients with IBD^45^. In an IBD mouse model, such as DSS- or 2,4,6-trinitrobenzenesulfonic acid (TNBS)-treated mice, the inhibition of the TGF-β_1_/Smad pathway reduced intestinal fibrosis^46,47^. Kashima *et al*. reported that *Lactobacillus brevis*-derived polyphosphate downregulated the TGF-β_1_/Smad pathway to affect both the intestinal epithelia and macrophages^46^. Li *et al*. also reported that pirfenidone, an antifibrotic agent for idiopathic pulmonary fibrosis, inhibited fibroblast proliferation and TGF-β signaling^47^. We found that MA-35 reduced the fibrotic cytokines *Tgfb1* and *Fn1* and intestinal fibrosis by inhibiting the TGF-β_1_/Smad pathway in an AOM/DSS mouse model. In this scenario, MA-35 could be a potential therapeutic drug candidate for fibrosis, not just anti-inflammation and antitumorigenic effects.

Recent studies reported that various anti-TNF-α antibodies, such as Infliximab, inhibited tumorigenesis in the AOM/DSS mouse model^14^. Compared to this antibody injection therapy, MA-35 shows sufficient efficacy by oral administration. Furthermore, the dual blockade of TNF-α signaling and TGF-β_1_ signaling attenuated both the development of CAC and fibrosis and had a beneficial effect against inflammation and fibrosis in various diseases.

Various flavonoids were shown to attenuate inflammation and prevent AOM/DSS-induced carcinogenesis. For example, black raspberry anthocyanins prevented colon cancer by modulating the composition of gut commensal microbiota and the methylation status of the SFRP2 gene^48^. Nobiletin isolated from citrus peel also inhibited colitis-associated cancer by downregulating iNOS, inducing antioxidative enzymes and arresting cell cycle progression^49^. Furthermore, isorhamnetin, a metabolite quercetin, prevented colon cancer by inhibiting oncogenic Src activity and β-catenin nuclear translocation^50^. Although there are divergent mechanisms that attenuate inflammation and prevent AOM/DSS-induced carcinogenesis, our data suggested that MA-35 could be an additional option for inhibiting colitis-associated cancer and fibrosis by inhibiting both the TNF-α signaling and TGF-β_1_ signaling pathways.

In conclusion, MA-35 is a potent candidate that inhibits the development of CAC by reducing inflammation and fibrosis in an AOM/DSS mouse model by inhibiting both TNF-α signaling and TGF-β_1_ signaling.

## Methods

### Cell culture and treatments

The human colon cancer cell line HT-29 (ATCC HTB-38) was obtained from ATCC (Manassas, VA). HT-29 cells were cultured in McCoy’s 5 A medium containing 10% fetal bovine serum (FBS) and 1% penicillin-streptomycin (PS). In Western blot experiments, each cell type was incubated in serum-free medium for 24 h prior to each stimulation. After pretreatment with MA-35 at a dose of 10 μM, 30 μM, or 50 μM for 60 min, recombinant human TNF-α (10 ng/ml, PeproTech, Rocky Hill, NJ) or recombinant human TGF-β_1_ (10 ng/ml, PeproTech) was added to the culture for various periods of time.

### Animal studies

All animal experiments were approved by the Tohoku University Animal Care Committee. The experimental protocols and animal care were performed according to the guidelines for animal experiments at Tohoku University. Male ICR mice aged 5 weeks were purchased from CLEA Japan, Inc., fed common commercial pellet diets and ordinary tap water and housed in an air-conditioned room at a temperature of 24°C. A colitis-associated cancer model mouse was made by intraperitoneal injection of 10 mg/kg AOM (Wako, Tokyo, Japan) and by oral administration of 2.5% DSS with a molecular weight of 36,000–50,000 (MP Biomedicals, Santa Ana, OH). Five-week-old male ICR mice were acclimatized for the first week. At 6 weeks of age, mice were randomly divided into a control group (n = 8), AOM/DSS group (n = 8) and AOM/DSS + MA-35 group (n = 8). In the AOM/DSS-treated group, mice were injected intraperitoneally with AOM (10 mg/kg body weight). Seven days later, 2.5% DSS was given in the drinking water over 7 days, followed by 14 days of regular water. Three cycles of DSS treatment were repeated. MA-35 was orally administered every 24 h for 70 days with a feeding tube. All mice were sacrificed at the end of the third cycle, and blood samples and colon were obtained. We measured the colon length between the ileocecal junction and rectum. Each colon was cut open longitudinally, and the normal tissue of the proximal colon, dysplasia tissue of the middle colon and cancer tissue of the distal colon were distinguished and assessed using a stereoscopic microscope^33^. Sections of these normal, dysplasia, and cancer tissues were stored in liquid nitrogen for quantitative real-time RT-PCR, fixed in 10% buffered formalin and embedded in paraffin for histological examination. Biochemical parameters were measured by a blood analyzer (i-STAT, Fuso Pharmaceutical Industries, Osaka, Japan). The serum AST level was measured with Fuji DRI-CHEM SLIDE GOT/AST-PIII (Fujifilm, Tokyo, Japan), and the serum ALT level was measured with Fuji DRI-CHEM SLIDE GPT/ALT-PIII (Fujifilm).

### Clinical disease score

Mice were monitored twice a week for body weight, stool consistency and stool bleeding. Colitis severity was scored by evaluating these clinical disease activities. The Disease Activity Index (DAI) was determined as previously described as follows^51^; change in body weight loss (no weight loss or weight gain = 0; 5–10% weight loss = 1; 11–15% weight loss = 2; 16–20% weight loss = 3; >21% weight loss = 4), stool consistency (normal and well-formed = 0; very soft and unformed = 2; watery stool = 4) and stool bleeding (normal color stool = 0; reddish color stool = 2; bloody stool = 4). The DAI score was calculated as the total of these scores and ranged from 0 (healthy) to 12 (severe colitis).

### Calculation of cancer area in the colon

After sacrifice, the protruded area (which may have included cancer) in the colon was macroscopically confirmed. All colons were photographed, and the pictures were stored on the computer. Then, the protruded area and whole colon area were measured by NIH ImageJ software, and the percentage of the protruded area was calculated as follows: the protruded area/the area of whole colon × 100.

### Histological examination

The colon was fixed in 10% buffered formalin, embedded in paraffin, and then stained with hematoxylin and eosin (H-E) and Masson’s trichrome stain (MTS). To evaluate inflammation, H-E-stained colonic tissue sections were scored using the following measures^31,32^: crypt architecture (normal = 0: severe crypt distortion with loss of entire crypt = 3), degree of inflammatory cell infiltration (normal = 0; dense inflammatory infiltrate = 3), muscle thickening (base of crypt sits on the muscularis mucosae = 0; marked muscle thickening present = 3), goblet cell depletion (absent = 0; present = 3) and crypt abscess (absent = 0; present = 3). The histological damage score is the sum of each individual score. We scored from the middle colon to proximal colon at ×20 objective, and the maximal score was used for evaluation.

To evaluate fibrosis of colon, the percentage of the positive area of MTS was quantified by measurement at ×20 objective in 5 randomly non-overlapping fields from the middle colon to proximal colon using ImageJ software.

### Reverse transcription and quantitative real-time PCR

Total RNA was extracted using the RNeasy Mini Kit (Qiagen, Hilden, Germany) according to the manufacturer’s instructions. cDNA was synthesized using the PrimeScript RT reagent Kit (TaKaRa Bio, Shiga, Japan). Quantitative real-time PCR was performed using the TaqMan Gene Expression Assay (Applied Biosystems, Foster City, CA) according to the manufacturer’s instructions using a StepOnePlus Real Time PCR System (Applied Biosystems). The primers used are listed in Table 2. The cycle threshold (Ct) was calculated using the comparative CT method (ddCT method). Relative mRNA expression was normalized to GAPDH.

**Table 2:** Primer list used in PCR analysis.

Taqman Gene Expression Assays (mouse)	
*Fn1*	Mm01256744_m1
*Tgfb1*	Mm01178820_m1
*Tnfa*	Mm00443260_m1
*Ccl2*	Mm00441242_m1
*Cd68*	Mm03047343_m1
*Il6*	Mm00446190_m1
*GAPDH*	Mm99999915_m1

### Western blot analysis

Equal amounts of protein were separated by SDS-PAGE on a 10% gel. Proteins were transferred onto a polyvinylidene difluoride membrane. The membrane was incubated overnight with primary antibodies against p-IKK, IKK, p-Smad3, Smad3, p-Smad2, Smad2, p-NF-κB p65, NF-κB p65, TNF-α, TGF-β (all from Cell Signaling Technology (CST), Danvers, MA), and Fn1 (from Abcam, Cambridge, UK) and incubated for 1 h with HRP-conjugated secondary antibodies (CST). Protein bands were detected using the enhanced chemiluminescent plus system with Clarity Western ECL Substrate (Bio-Rad, Hercules, CA). Band intensities were analyzed by NIH ImageJ software.

### Statistical analyses

All data are presented as the mean ± SEM. Statistical analysis was evaluated by Student’s *t*-test or analysis of variance (ANOVA) followed by Dunnett’s multiple comparison test and Tukey’s test. The survival rate was evaluated by log-rank test using the Kaplan-Meier method. Values of *p* < 0.05 were considered statistically significant. JMP Pro software version 14 (SAS Institute Inc., Cary, NC) was used for statistical analysis.

## Supplementary information

Supplementary Figure1 & 2
