# Supplementary material for: The indole compound MA-35 attenuates tumorigenesis in an inflammation-induced colon cancer model
Source: Sci Rep. 2019 Sep 4;9:12739. doi: 10.1038/s41598-019-48974-9 (PMC6726640; doi:10.1038/s41598-019-48974-9)
Supplement: Supplementary file 1 — Supplementary Figure1 & 2 [file 41598_2019_48974_MOESM1_ESM.pdf]

Supplementary information

An indole compound MA-35 attenuates tumorgenesis in an inflammation-induced colon cancer model

Keigo Kanehara[1], Shinobu Ohnuma[1], Yoshitake Kanazawa[2], Keisuke Sato[1], Shouji Kokubo[1], Hideyuki Suzuki[1], Hideaki Karasawa[1], Takehiro Suzuki[3], Chitose Suzuki[3], Takeshi Naitoh[1], Michiaki Unno[1] and Takaaki Abe[3,4,5,]

1. Department of Surgery, Tohoku University Graduate School of Medicine, Sendai 980-8574, Japan
2. Department of Gastroenterology, Tohoku University Graduate School of Medicine, Sendai 980-8574, Japan
3. Division of Nephrology, Endocrinology, and Vascular Medicine, Tohoku University Graduate School of Medicine, Sendai 980-8574, Japan
4. Department of Medical Science, Tohoku University Graduate School of Biomedical Engineering, Sendai 980-8574, Japan
5. Department of Clinical Biology and Hormonal Regulation, Tohoku University Graduate School of Medicine, Sendai 980-8574, Japan

¶To whom correspondence should be addressed:
Takaaki ABE M.D., Ph.D.
e-mail: takaabe@med.tohoku.ac.jp

Supplementary Figure 1

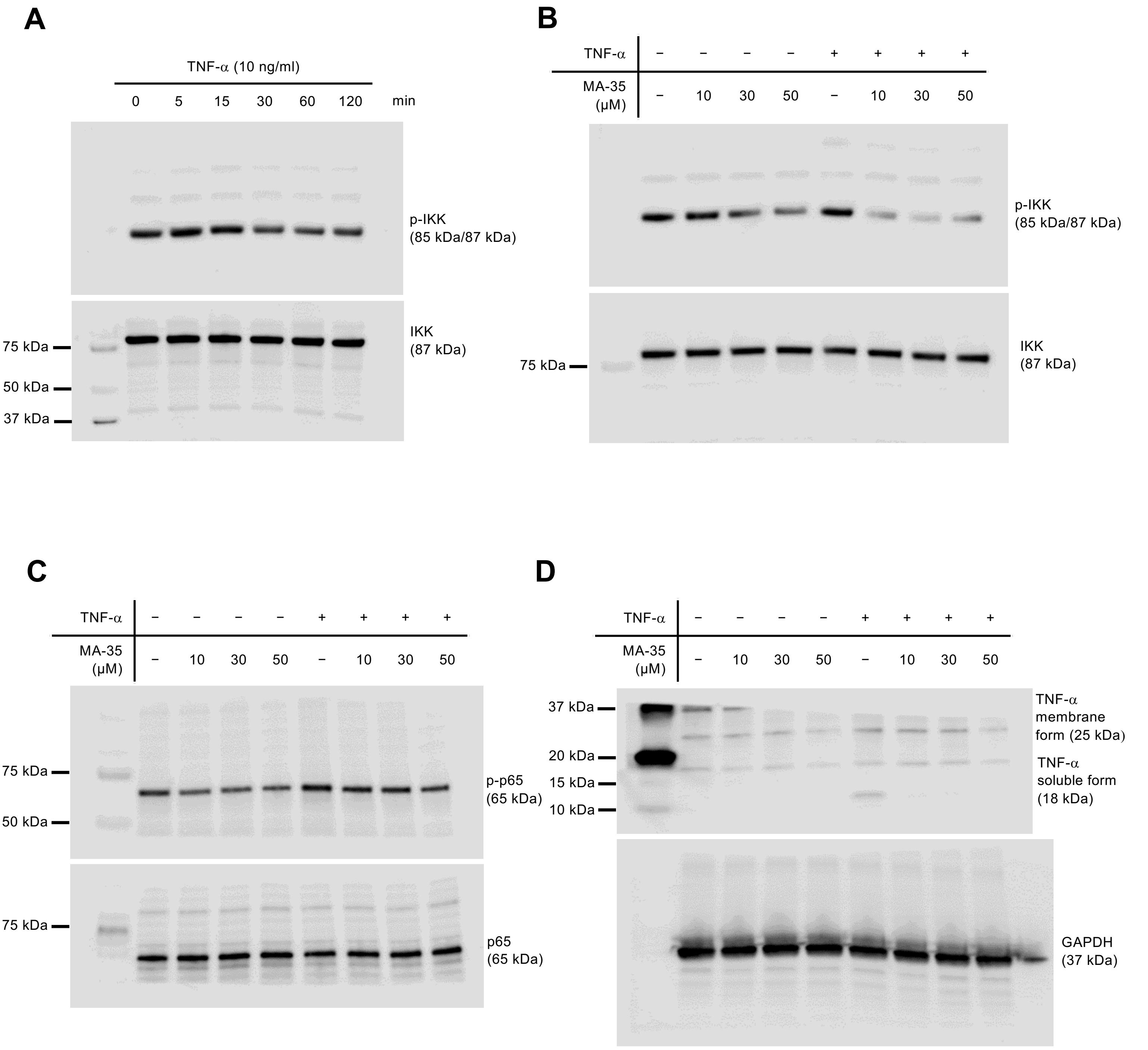

Supplementary Figure 1. Raw data of the western blotting experiments presented in Figure 5. In all cases the lanes are displayed over entire length of the membrane. The blotting membrane was stripped before incubation with anti-p-IKK antibody, anti-IKK antibody, anti-p-p65 antibody, anti-p65 antibody, anti-TNF-α antibody and anti-GAPDH antibody.

Supplementary Figure 2

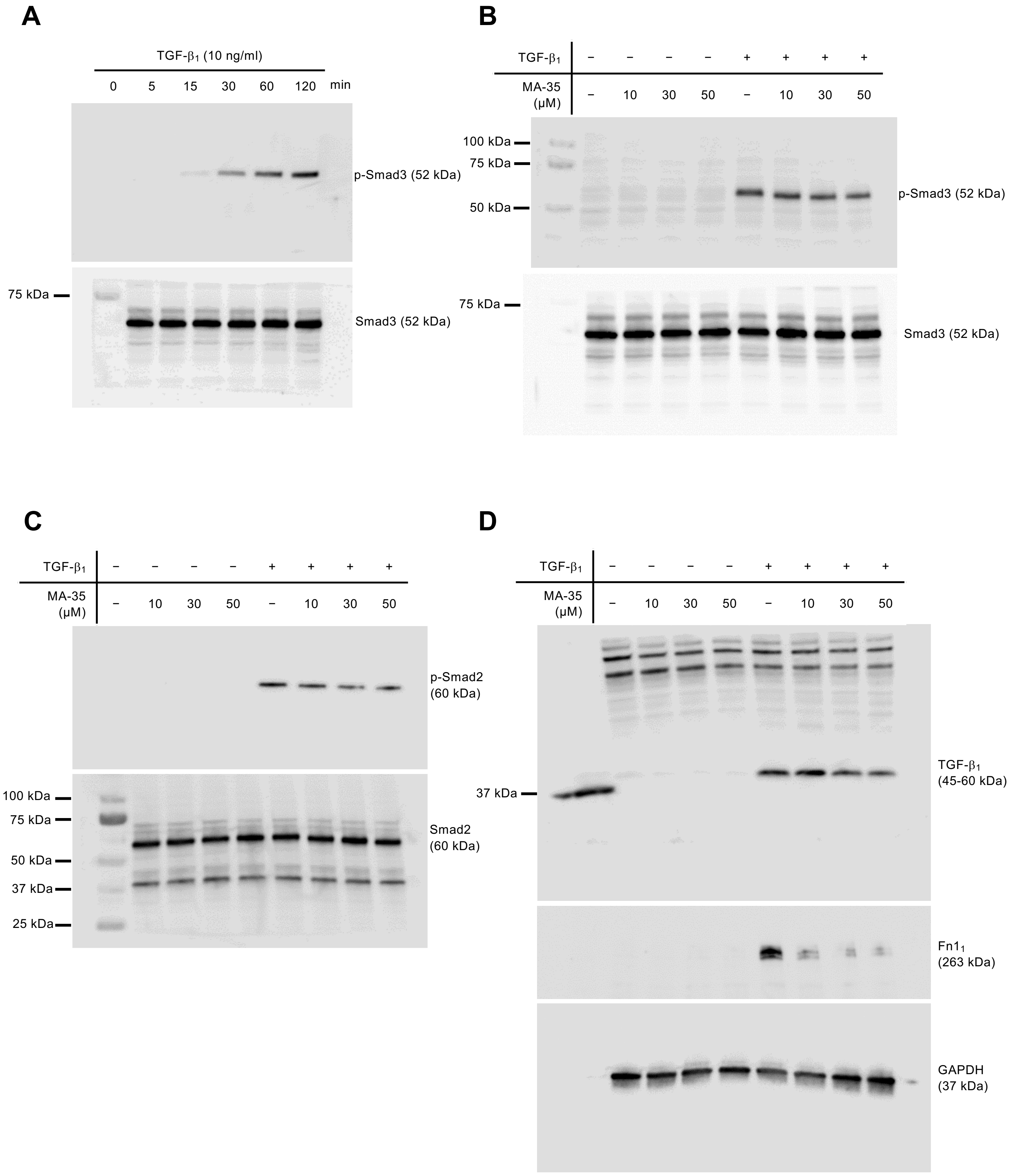

Supplementary Figure 2. Raw data of the western blotting experiments presented in Figure 6. In all cases the lanes are displayed over entire length of the membrane. The blotting membrane was stripped before incubation with anti-p-Smad3 antibody, anti-Smad3 antibody, anti-p-Smad2 antibody, anti-Smad2 antibody, anti-TGF-β_1 antibody, anti-Fn1 antibody and anti-GAPDH antibody .
